# Comparative study on nutrient composition, phytochemical, and functional characteristics of raw, germinated, and fermented *Moringa oleifera* seed flour

**DOI:** 10.1002/fsn3.70

**Published:** 2013-10-21

**Authors:** Oluwole S Ijarotimi, Oluwole A Adeoti, Oluwaseun Ariyo

**Affiliations:** 1 Department of Food Science and Technology, Federal University of TechnologyAkure, Nigeria; 2 Department of Human Nutrition, Faculty of Public Health, College of Medicine, University of IbadanIbadan, Oyo State, Nigeria

**Keywords:** Functional properties, *Moringa oleifera* seed, nutrient composition, phytochemical/antinutrients

## Abstract

*Moringa oleifera* seeds were processed as raw *M. oleifera* (RMO), germinated *M. oleifera* (GMO), and fermented *M. oleifera* (FMO), and were evaluated for proximate, minerals, amino acids, fatty acids, phytochemicals/antinutrients, and functional properties. Protein content of GMO (23.69 ± 0.11 g/100 g) was higher than FMO (21.15 ± 0.08 g/100 g) and that of RMO (18.86 ± 0.09 g/100 g) (*P* < 0.05), respectively. Energy value of FMO (465.32 ± 0.48 kcal) was higher than GMO (438.62 ± 0.12 kcal) and that of RMO (409.04 ± 1.61 kcal), respectively. Mineral contents in GMO were significantly higher in iron, sodium, potassium, magnesium, and copper, while FMO were higher in calcium, phosphorus, and magnesium, and both were significantly lower than those in RMO (*P* < 0.05). Total essential amino acids (TEAAs) in FMO (31.07 mg/g crude protein) were higher than in GMO (26.52 mg/g crude protein), and were higher than that in RMO (23.56 mg/g crude protein). Linoleic acid (58.79 ± 0.02–62.05 ± 0.01 g/100 g) and behenic acid (0.13 ± 0.00–0.20 ± 0.06 g/100 g) were the predominant and least fatty acids, respectively. Phytochemical/antinutrient compositions in FMO samples were significantly lower than GMO, and both were significantly lower when compared with RMO samples (*P* < 0.05). The bulk density (pack and loose), foaming capacity, swelling capacity, and water absorption capacity (WAC) of FMO were significantly higher than those of GMO, and there was no significant difference between GMO and RMO samples. The study established that fermentation processing methods increased the protein content, essential amino acid, and polyunsaturated fatty acid profiles, and reduced antinutrient compositions of *M. oleifera* seed than germination processing techniques; hence, fermentation techniques should be encouraged in processing moringa seeds in food processing.

## Introduction

*Moringa oleifera* Lam. is a type of vegetable plant shrub 5–15 m in height and with soft and brittle stems (Roloff et al. 2009) with a diameter of about 30 cm. Moringa leaves are compound, pinnate double, and of small round or oval shape. The fruit, called “drumstick,” is long and angular, its sides form a triangle; the drumsticks are about 15–45 cm-long, with around 20 (Sengupta and Gupta 1970) seeds. Moringa grow well in the humid tropics or hot dry lands and can survive in less fertile soils and it is also little affected by drought (Anwar et al. 2007). Moringa is native to the Indian subcontinent and has become naturalized in the tropical and subtropical areas around the world. The tree is known by such regional names as Benzolive, Drumstick tree, Horseradish tree, Kelor, Marango, Mlonge, Mulangay, Saijihan, and Sajna (Fahey 2005). The plant is considered as one of the world's most useful trees, as almost every part of the Moringa tree can be used for food, medication, and industrial purposes (Khalafalla et al. 2010). People use its leaves, flowers, and fresh pods as vegetables, while others use it as livestock feed (Anjorin et al. 2010). This tree has the potential to improve nutrition, boost food security, and foster good health status (Hsu 2006). Recently, the utilization of the plant is increasing in most countries where it originated and in non-native (Reyes Sanchez et al. 2006; Oduro et al. 2008) countries, due to its nutritional, therapeutic, and prophylactic properties (Fahey 2005).

Moringa is considered to be the most nutrient-rich plant on earth. Moringa leaves have been consumed by Asian people for millennia as a healthy food product. Studies from other countries indicate that the leaves have immense nutritional value such as phytochemicals, vitamins, minerals, and amino acids (Anwar et al. 2007; Busani et al. 2011). As such, the leaves have been used to combat malnutrition, especially among infants and nursing mothers. The Romans, Greeks, and Egyptians extracted edible oil from the seeds and used it for perfume and as a skin lotion. People in the Indian subcontinent have long used Moringa pods for food. The edible leaves are eaten throughout West Africa and parts of Asia. Moringa leaves are edible and are of high nutritive value (Waldron et al. 2003; Tetteh 2008). It has been reported that *M. oleifera* leaf product, especially leaf powder, is becoming increasingly popular in Nigeria because of its outstanding indigenous nutritive and medicinal value. The leaves are also free of antinutritive factors such as phenols, tannins, and saponins (Fuglie 2001).

Moringa seeds have long been used by the public as a tasty vegetable and water purifier because of its coagulant properties (Ayotunde et al. 2011). Other moringa plant parts like flowers, roots, and bark also have good nutritional and therapeutic value (Olushola 2006). The purpose of this study was to investigate the effects of germination and fermentation on the chemical, bioactive, and functional characteristics of moringa seed flour.

## Material and Methods

### Procurement of materials

*Moringa oleifera* seeds were collected from Makurdi in January 2012, where it is commonly grown.

### Processing of *M. oleifera* seed flour

**Figure 1:**
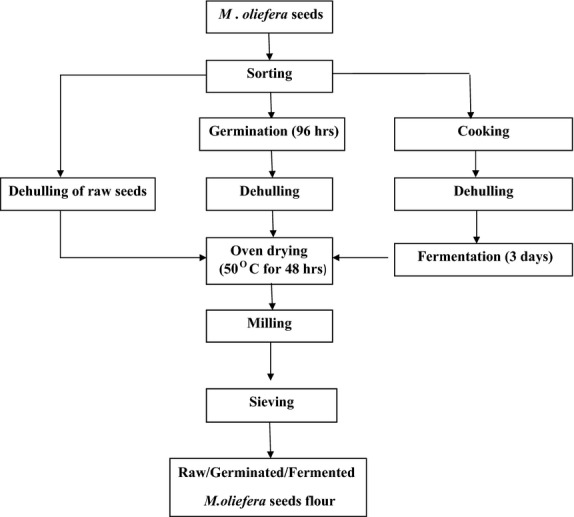
Processing of raw, germinated, and fermented *Moringa oleifera* flour samples.

*Moringa oleifera seeds* were processed into raw, germinated, and fermented flour as described below:

*Raw M. oleifera seed flour*: Raw *M. oleifera* seeds were sorted, pretreated for 5 min with 200 ppm of bleach containing 5.25% sodium hypochlorite, and mixed in deionized water to control microbial growth. Seeds were rinsed, soaked in deionized water (1:3, w/v) for 9 h at ambient temperature (23–25°C), dehulled, and ovendried at 50°C (Plus11; Sanyo Gallenkamp PLC, Leicestershire, U.K.) for 20 h, milled using a Philips laboratory blender (HR2811 model) and sieved using a 60-mm mesh sieve (British Standard) to obtain RMO seed flour. The flour was packed in a plastic container sealed with an aluminum foil and stored at room temperature (˜27°C) until required for use (Fig. 1).
*Germinated M. oleifera seed flour*: *Moringa oleifera* seeds were sorted, pretreated for 5 min with 200 ppm of bleach containing 5.25% sodium hypochlorite, and mixed in deionized water to control microbial growth. Seeds were rinsed and soaked in deionized water (1:3, w/v) for 9 h at ambient temperature (23–25°C). Seeds were drained and placed on perforated aluminum pans lined with filter paper, then placed in a dark, temperature-controlled cabinet at 30°C for germination. After 4 days, the seeds were germinated and the germinated seeds were washed with distilled water manually, ovendried at 50°C (Plus11; Sanyo Gallenkamp PLC) for 20 h, milled using a Philips laboratory blender (HR2811 model), and sieved using a 60-mm mesh sieve (British Standard). The popcorn flour was packed in a plastic container sealed with an aluminum foil and stored at room temperature (27°C) prior to analyses (Fig. 1).
*Fermented M. oleifera seed flour*: *Moringa oleifera* seeds were sorted, dehulled, boiled for 1 h as described by Bakebain and Giami (1992), wrapped in blanched banana leaves at room temperature for 72 h to ferment. The fermented seeds were ovendried at 50°C (Plus11; Sanyo Gallenkamp PLC) for 20 h, milled using a Philips laboratory blender (HR2811 model), and sieved using a 60-mm mesh sieve (British Standard). The popcorn flour was packed in a plastic container sealed with an aluminum foil and stored at room temperature (27°C) prior to analyses.

### Proximate composition determination using methods of Association of Analytical Chemists

#### Moisture content

One gram of sample in pre-weighed crucible was placed in an oven (105°C) for 24 h, cooled, and reweighed. The percentage moisture was calculated as follows:


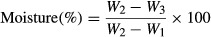


where *W*1 is the weight of the crucible, *W*2 is the weight of the crucible after drying at 105°C and sample, and *W*3 is the weight of the crucible and the sample after cooling in airtight desiccators.

#### Crude protein

Crude protein content was determined using the micro-Kjeldahl method as described by Pearson (1976). A volume of 10 mL H_2_SO_4_ added to 3 g of sample was digested with a Kjeldahl digestor (Model Bauchi 430) for 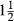 h. A volume of 40 mL water was added and distilled using a Kjeldahl distillation Unit (Model unit B – 316) containing 40% concentrated sodium hydroxide and Millipore water. Liberated ammonia was collected in 20 mL boric acid with bromocresol green and methyl red indicators and titrated against 0.04 N H_2_SO_4_. A blank (without sample) was likewise prepared. Percent protein was calculated as:


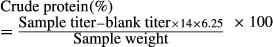


where 14 is the molecular weight of nitrogen and 6.25 is the nitrogen factor.

#### Crude fiber

A weighed crucible containing 1 g of defatted sample was attached to the extraction unit (in Kjeldahl, D-40599; Behr Labor-Technik GmbH, Dusseldorf, Germany) and into this 150 mL of hot 1.25% H_2_SO_4_ was added and digested for 30 min, the acid was drained and sample washed with hot distilled water for 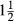 h. The crucible was removed and ovendried overnight at 105°C, cooled, weighed, and incinerated at 550°C in a muffle furnace (MF-1-02; PCSIR Labs, Lahore, Pakistan) overnight and reweighed after cooling. Percentage extracted fiber was calculated as:


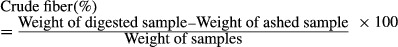


#### Lipid

Lipid content was estimated using TecatorSoxtec (Model 2043[20430001]; Hilleroed, Denmark). A quantity of 1.5 g sample mixed with 2.3 g anhydrous sulfate was weighed into a thimble and covered with absorbent cotton, while 40 mL of petroleum ether (40–60°C Bpt) was added to a pre-weighed cup. Both thimble and cup were attached to the Extraction Unit. The sample was extracted using ethanol for 30 min and rinsed for 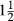 h. Thereafter, the solvent was evaporated from the cup to the condensing column. Extracted fat in the cup was then placed in an oven at 105°C for 1 h and cooled and weighed. Percent fat was calculated as:


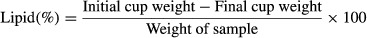


#### Ash

Ash and mineral contents were determined according to AOAC (Association of Analytical Chemists) numbers 923.03 and 984.27 (AOAC 2005). Two grams of sample was added into a pre-weighed crucible was incinerated in muffle furnace at 600°C.


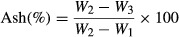


where *W*1 is the weight of cleaned, dried, ignited, and cooled crucible, *W*2 the weight of the crucible and sample after incinerating at 600°C, and *W*3 the weight of the crucible and sample after cooling in an airtight homogenized vessel.

#### Carbohydrate

The carbohydrate content was determined by difference, that is, addition of all the percentages of moisture, fat, crude protein, ash, and crude fiber was subtracted from 100%. This gave the amount of nitrogen-free extract otherwise known as carbohydrate.


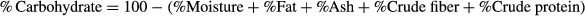


#### Energy value

The sample energy value was estimated (in kcal/g) by multiplying the percentages of crude protein, crude lipid, and carbohydrate with the recommended factors (2.44, 8.37, and 3.57, respectively) as proposed by Martin and Coolidge (1978).

### Mineral determination

AOAC (2005) methods were used to determine the mineral compositions of the samples. One gram of sample was digested with nitric/perchloric/sulfuric acid mixture in the ratio 9:2:1, respectively, and filtered. The filtrate was made up to mark in a 5-mL volumetric flask. The filtered solution was loaded to an Atomic Absorption Spectrophotometer (model 703; Perkin Elmes, Norwalk, CT). The standard curve for each mineral, that is, calcium, magnesium, iron, aluminum, lead, copper, and zinc, was prepared from known standards and the mineral value of samples estimated against that of the standard curve. Values of sodium and potassium were determined using a Flame photometer (Sherwood Flame Photometer 410; Sherwood Scientific Ltd., Cambridge, U.K.) using NaCl and KCl as the standard (AOAC 2005), while phosphorus was determined using the Vanodo-molybdate method.

### Determination of amino acid profile

Moringa seed flour sample hydrolysates were prepared following the method of Spackman et al. (1958). Each of the defatted samples was weighed (200 mg) into a glass ampoule, 5 mL of 6 mol/L HCl was added to this, and the contents were hydrolyzed in an oven preset at 105 ± 5°C for 22 h. Oxygen was expelled in the ampoule by passing nitrogen gas into it. Amino acid analysis was done by ion-exchange chromatography (Spackman et al. 1958) using a Technicon Sequential Multisample Amino Acid Analyzer (Technicon Instruments Corporation, New York, NY). The period of analysis was 76 min, with a gas flow rate of 0.50 mL/min at 60°C, and the reproducibility was ±3%. The amino acid composition was calculated from the areas of standards obtained from the integrator and expressed as percentages of the total protein.

### Determination of fatty acids of *M. oleifera* seed flour samples

Fatty acid compositions of the samples were analyzed using gas-liquid chromatography (with omega-wax capillary column Supelco, Sigma-Aldrich, Bellefonte, PA). The lipid classes were separated by thin layer chromatography on silica gel G 60 (Merck, Darmstadt, Germany), using *n*-hexane/ethyl ether/acetic acid (73/25/2/v/v/v) as developing solvent. The fatty acids of phospholipids and triglycerides were transformed with sodium methylate into methyl esters.

### Determination of bioactive and antinutritional composition of *M. oleifera* samples

#### Determination of alkaloid

Determination of alkaloid was made by the method described by Harborne (1973). The alkaloid content was determined gravimetrically. Five grams of the sample was weighed and dispersed in 10% acetic acid solution in ethanol to form a ratio of 1:10 (10%). The mixture was allowed to stand for 4 h at 28°C. It was later filtered via Whatman No. 42 grade of filter paper. The filtrate was concentrated to one quarter of its original volume by evaporation and treated with drop wise addition of concentrated aqueous NH_4_OH until the alkaloid was precipitated. The alkaloid precipitated was received in a weighed filter paper, washed with 1% ammonia solution, and dried in the oven at 80°C. Alkaloid content was calculated and expressed as a percentage of the weight of sample analyzed.

#### Determination of saponins

The spectrophotometric method was used for saponin analysis as described by Brunner (1984). One gram of the flour sample was weighed into a 250-mL beaker and 100 mL isobutyl alcohol was added. The mixture was shaken on a UDY shaker (UDY Corporation, Fort Collins, CO) for 5 h to ensure uniform mixing. The mixture was filtered through a Whatman No. 1 filter paper into a 100-mL beaker and 20 mL of 40% saturated solution of magnesium carbonate was added. The mixture obtained was further filtered through a Whatman No. 1 filter paper to obtain a clear colorless solution. One milliliter of the colorless solution was homogenized into a 50-mL volumetric flask and 2 mL of 5% FeCl_3_ solution was added and made up to mark with distilled water and allowed to stand for 30 min for blood red color to develop. Standard saponin solutions (0–10 ppm) were prepared from saponin stock solution and treated with 2 mL of 5% FeCl solution as done for experimental samples. The absorbance of the sample as well as standard saponin solutions were read after color development on a Spectronic 2lD spectrophotometer (Milton Roy, Houston, TX) at a wavelength of 380 nm. The percentage saponin was also calculated.

#### Determination of total phenolic compounds

The samples (100 g) were extracted, by stirring with methanol 250 mL for 3 h. The extracted samples were then filtered through Whatman No. 1 filter paper, the residue was washed with 100 mL methanol, and the extracts were cooled. The extracts were evaporated to dryness under vacuum, using a rotary evaporator. The residues were dissolved with 10 mL of methanol and used for determination of total phenolic compounds. This determination was performed as gallic acid equivalents (mg/100 g), by using Folin-Ciocalteau phenol reagent. The diluted methanol extracts (0.2 mL) were added, with 0.8 mL of Folin-Ciocalteau phenol reagent and 2.0 mL of sodium carbonate (7.5%), in the given order. The mixtures were vigorously vortex-mixed and diluted to 7 mL of deionized water. The reaction was allowed to complete for 2 h in the dark, at room temperature, prior to being centrifuged for 5 min at 125 g. The supernatant was measured at 756 nm on a spectrophotometer. Methanol was applied as a control, by replacing the sample. Gallic acid was used as a standard and the results were calculated as gallic acid equivalents (mg/100 g) of the sample. The reaction was conducted in triplicate and the results were averaged.

#### Determination of total flavonoid

This was also determined according to the method outlined by Harborne (1973). Five grams of the sample was boiled in 50 mL of 2 mol/L HCl solution for 30 min under reflux. The contents were allowed to cool and then filtered through a Whatman No. 42 filter paper. A measured volume of the extract was treated with equal volume of ethyl acetate starting with a drop. The flavonoid precipitated was recovered by filtration using weighed filter paper. The resulting weight difference gave the weight of flavonoid in the sample.

#### Determination of tannin content

Tannin content of the flour samples was determined using the methods described by Swain (1979). The sample (0.2 g) was measured in a 50-mL beaker; 20 mL of 50% methanol was added, covered with homogenizer, placed in a water bath at 77–80°C for 1 h, and the contents stirred with a glass rod to prevent lumping. The mixture was filtered using a double-layered Whatman No. 1 filter paper into a 100-mL volumetric flask using 50% methanol to rinse. This was made up to mark with distilled water and thoroughly mixed. One milliliter of the sample extract was homogenized into a 50-mL volumetric flask, and 20 mL distilled water, 2.5 mL Folin-Denis reagent, and 10 mL of 17% Na_2_CO_3_ were added and mixed. The mixture was made up to mark with distilled water, thoroughly mixed, and allowed to stand for 20 min when a bluish-green coloration developed. Standard tannic acid solutions in the range of 0–10 ppm were treated similarly as the 1 mL sample above. The absorbances of the tannic acid standard solutions as well as samples were read after color development on a Spectronic 21D spectrophotometer at a wavelength of 760 nm. Percentage tannin was calculated.

#### Determination of phytic acid

An indirect colorimetric method of Wheeler and Ferrel (1971) was used for phytate determination. This method depends on an iron to phosphorus ratio of 4:6. A quantity of 5 g of the test sample was extracted with 3% trichloro acetic acid. The phytate was precipitated as ferric phytate and converted to ferric hydroxide and soluble sodium phytate by adding sodium hydroxide. The precipitate was dissolved in hot 3.2 N HNO and the color read immediately at 480 nm. The standard solution was prepared from Fe(NO_3_)_3_ and the iron content was extrapolated from a Fe(NO)_3_ standard curve. The phytate concentration was calculated from the iron results assuming a 4:6 iron:phosphorus molecular ratio.

#### Determination of oxalate content

Oxalate was determined by AOAC (2005) method. One gram of the sample was weighed in a 100-mL conical flask. Seventy-five milliliters of 3 mol/L H_2_SO_4_ was added and the solution was stirred intermittently with a magnetic stirrer for about 1 h and then filtered using Whatman No. 1 filter paper. The sample filtrate (extract) (25 mL) was collected and titrated against hot (80–90°C) 0.1 N KMnO_4_ solution to the point when a faint pink color appeared that persisted for at least 30 sec. The concentration of oxalate in each sample was obtained from the calculation: 1 mL 0.1 permanganate = 0.006303 g oxalate.

### Functional properties

#### Water absorption capacity

Water and oil absorption capacities of the flour samples were determined by Beuchat (1977) methods. Each of the formulated samples was weighed (20 g) and hydrated with 100 mL of distilled water at 25°C for 1 h with manual stirring at 10-min intervals. The excess water was drained with a Whatman No. 2 filter paper with slight suction. The water absorption index was calculated as follows:


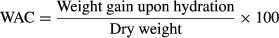


#### Bulk density

A 50 g flour sample was put into a 100-mL measuring cylinder. The cylinder was tapped continuously until a constant volume was obtained. The bulk density (g cm^−3^) was calculated as weight of flour (g) divided by flour volume (cm^3^) (Okaka and Potter 1979).

#### Swelling capacity

This was determined using the method described by Leach et al. (1959) with modification for small samples. One gram of the flour sample was mixed with 10 mL distilled water in a centrifuge tube and heated at 80°C for 30 min. This was shaken continuously during the heating period. After heating, the suspension was centrifuged at 1000*g* for 15 min. The supernatant was decanted and the weight of the paste taken. The swelling power was calculated as:


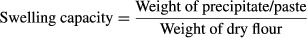


#### Foaming properties

Foaming capacity and stability were determined according to the method reported by Coffman and Garcia (1977). Two grams of seed flour was whipped (homogenized) with 100 mL distilled water for 15 min at speed setting max and then poured into a 250-mL graduated measuring cylinder. The total volume at time intervals of 0.0, 0.25, 3.00, and 4.00 h was noted. Percent volume increase was calculated as follows:


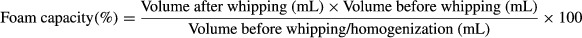



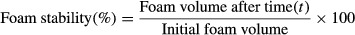


### Statistical analysis

The data were analyzed using SPSS version 15.0 (SPSS Inc., Chicago, IL). The mean and standard error of means (SEM) of the triplicate analyses of the samples were calculated. Analysis of variance was performed to determine significant differences between the means, while the means were separated using the new Duncan multiple range test and *P* < 0.05 was applied to establish significant differences.

## Results and Discussion

### Proximate composition of *M. oleifera* seed flour

Proximate compositions of RMO, GMO, and FMO seed flour are presented in Table 1. The moisture contents (MS) of FMO seed flour (8.13 ± 0.09 g/100 g) was significantly lower when compared with GMO seed flour (9.43 ± 0.03 g/100 g), and RMO seed flour (10.60 ± 0.06 g/100 g) samples, respectively (*P* < 0.05). The observed low MS in the processed seed flour could be due to the effect of processing techniques. The lower MS observed in this study is an indication that the activity of the microorganisms would be reduced and thereby increased the shelf life of the flour samples. This observation is in agreement with the report of Olitino et al. (2007). The protein content of GMO (23.69 ± 0.11 g/100 g) was significantly higher than that of the FMO (21.15 ± 0.08 g/100 g) seed flour samples, and RMO seed flour (18.86 ± 0.09 g/100 g) samples, respectively (*P* < 0.05). This observation was similar to the findings of other investigators, who reported that there were increases in the protein level of germinated and fermented cereals and legume-based food products compared with unprocessed products (Nnam 1995; Oshodi et al. 1999). The increased protein value of GMO and FMO seed flour could be attributed to the biochemical activities of the germinating seeds and the activities of the microorganisms during fermentation processing. Scientific studies have reported that during germination and fermentation carbohydrates are mobilized to synthesize amino acids for the growing seedling and proliferations of the microorganisms (Cronk et al. 1977; Abdelrahaman et al. 2007; Dubey et al. 2008; Ocheme and Chinma 2008; Ochanda et al. 2010). Crude fiber of the moringa seed flour ranged from 5.03 ± 0.07 g/100 g to 6.17 ± 0.03 g/100 g. The fiber content of moringa seed flour was quite low when compared with that of cowpea seeds reported by Mamiro et al. (2011). Dietary fiber, the indigestible cell wall component of plant materials, plays an important role in human health (Anderson 1990). A previous study linked low dietary fiber intake in developed countries with several Western diseases (Burkitt and Trowell 1975). Epidemiological studies have shown that high dietary fiber intake helps to prevent or treat hyperlipidemia (Anderson and Gustafson 1988), cardiovascular disease (Anderson 1990), hypertension (Anderson 1983), obesity (Anderson and Bryant 1986), certain cancers (Kromhout et al. 1982), gastrointestinal disorders (Madar and Odes 1990), and diabetes (Pilch 1987). The energy content of FMO sample (465.32 ± 0.48 kcal) was significantly higher than that of GMO flour sample (438.62 ± 0.12 kcal); however, both GMO and FMO energy values were comparatively higher than those of the RMO seed flour sample (409.04 ± 1.61 kcal; *P* < 0.05). This observation could be due to the increase in the fat content of germinated and fermented moringa seed flour when compared with that of the raw sample. This finding was similar to the report of Agbede (2001), who reported higher energy values for processed cowpea and some underutilized legumes.

**Table 1:** Proximate composition (mg/100 g) of raw, germinated, and fermented *Moringa oleifera* seed flour

Nutrient	RMO	GMO	FMO
Moisture (mg/100 g)	10.60 ± 0.06^a^	9.43 ± 0.03^b^	8.13 ± 0.09^c^
Protein (mg/100 g)	18.86 ± 0.09^c^	23.69 ± 0.11^a^	21.15 ± 0.08^b^
Fat (mg/100 g)	13.35 ± 0.03^c^	14.62 ± 0.04^a^	14.00 ± 0.09^b^
Ash (mg/100 g)	4.77 ± 0.04^a^	4.34 ± 0.04^c^	4.50 ± 0.04^b^
Fiber (mg/100 g)	5.03 ± 0.07^c^	5.48 ± 0.04^b^	6.17 ± 0.03^a^
Carbohydrate (mg/100 g)	53.36 ± 0.34^b^	53.00 ± 0.16^b^	61.07 ± 0.06^a^
Energy (kcal)	409.04 ± 1.61^c^	438.62 ± 0.12^b^	465.32 ± 0.48^a^

Values are means (±SEM) of triplicate samples. Means with different superscripts in the same row show significant difference (*P* < 0.05). RMO, raw *M. oleifera* flour; GMO, germinated *M. oleifera* flour; FMO, fermented *M. oleifera* flour.

### Mineral composition of *M. oleifera* seed flour

Table 2 shows the mineral composition of RMO, GMO, and FMO seed flour. Sodium was the most abundant mineral of the flour samples with a value range of 280.30 ± 0.03 mg/100 g for FMO to 295.10 ± 0.10 mg/100 g for RMO, while the least mineral was iodine with the value range of 0.10 ± 0.00 mg/100 g for FMO flour to 0.11 ± 0.01 mg/100 g for RMO. The Na/K and Ca/P ratios are indices of body electrolyte balance and bone formation and the values were quite high in this study. For instance, Na/K molar ratio range from 5.65 for RMO to 6.42 for FMO, while that of Ca/K molar ratio was Ca/P molar ratio was between 1.24 for RMO and 1.35 for GMO sample. The Na:K ratio <1 is recommended for diets, particularly for hypertensive patients. Therefore, the observed Na/K molar ratio of *M. oleifera* seed flour in this study may not be suitable for people who have the risk of high blood pressure. The high Ca/P ratio observed in this study is of nutritional benefit, particularly for children and the aged who need higher intakes of calcium and phosphorus for bone formation and maintenance. It is well known that diets with high value of Ca/P ratio are considered “good,” particularly for growing children who require high intake of calcium and phosphorus for bone and teeth formation (Nieman et al. 1992).

**Table 2:** Mineral composition (mg/100 g) of raw, germinated, and fermented *Moringa oleifera* seed flour

Minerals	RMO	GMO	FMO
Calcium	128.33 ± 1.67^a^	116.67 ± 1.67^b^	121.67 ± 1.67^b^
Phosphorus	103.33 ± 1.67^a^	86.67 ± 1.67^b^	91.67 ± 1.67^b^
Iron	7.33 ± 0.09^a^	6.63 ± 0.09^b^	5.63 ± 0.09^c^
Sodium	295.10 ± 0.10^a^	285.13 ± 0.13^b^	280.30 ± 0.03^c^
Potassium	52.33 ± 1.45^a^	45.00 ± 0.00^b^	43.67 ± 0.88^b^
Magnesium	26.33 ± 0.33^a^	25.10 ± 0.06^b^	25.13 ± 0.09^b^
Copper	0.63 ± 0.03^a^	0.60 ± 0.00^a^	0.57 ± 0.03^a^
Iodine	0.11 ± 0.01^a^	0.11 ± 0.01^a^	0.10 ± 0.00^a^
Na/K	5.65 ± 0.15^b^	6.33 ± 0.00^a^	6.42 ± 0.13^a^
Ca/P	1.24 ± 0.03^b^	1.35 ± 0.04^a^	1.33 ± 0.00^ab^
Ca/K	2.46 ± 0.09^b^	2.59 ± 0.04^ab^	2.79 ± 0.04^a^
Na/Mg	11.21 ± 0.14^a^	11.36 ± 0.03^a^	11.14 ± 0.04^a^
Ca/Mg	4.87 ± 0.06^a^	4.65 ± 0.07^a^	4.84 ± 0.08^a^
Fe/Cu	11.65 ± 0.69^a^	11.05 ± 0.15^a^	10.01 ± 0.61^a^

Values are means (±SEM) of triplicate samples; means with different superscripts in the same row show significant difference (*P* < 0.05). RMO, raw *M. oleifera* flour; GMO, germinated *M. oleifera* flour; FMO, fermented *M. oleifera* flour.

### Amino acid profiles of *M. oleifera* seed flour

Table 3 shows the amino acid profile of RMO, GMO, and FMO seed flour. The most abundant amino acid of moringa seed flour sample was glutamic acid with a value range from 17.87 ± 0.03 mg/g crude protein for RMO to 22.46 ± 0.06 mg/g crude protein for FMO, while valine was the least with a value range from 1.08 ± 0.01 mg/g crude protein for RMO to 1.64 ± 0.00 mg/g crude protein for FMO. The TEAA of FMO seed flour sample (20.815 g/100 g crude protein) was higher than that of GMO seed flour (17.653 g/100 g crude protein), and both were higher than that of the RMO seed flour sample (16.052 g/100 g crude protein). This observation indicates that germination and fermentation processing techniques increased the TEAA content of the flour samples. This finding is in agreement with that of other investigators, who reported that both germination and fermentation processing techniques increase the protein and amino acid profile of food samples (Cronk et al. 1977; Abdelrahaman et al. 2007; Dubey et al. 2008; Ocheme and Chinma 2008; Ochanda et al. 2010). On comparing with FAO/WHO (1990) recommended values, it was observed that the TEAAs in moringa samples were lesser. The amino acid scores of *M. oleifera* seed flour are presented in Table 4. Lysine and methionine were the first and second limiting amino acids in moringa seed flour, respectively.

**Table 3:** Amino acid (g/100 g crude protein) profile of raw, germinated, and fermented *Moringa oleifera* seed flour

Amino acids	RMO	GMO	FMO	FAO*
Nonessential amino acids				
Alanine	5.16 ± 0.01^c^	5.42 ± 0.00^b^	6.29 ± 0.00^a^	
Aspartic acid	15.70 ± 0.06^c^	18.13 ± 0.01^b^	21.37 ± 0.01^a^	
Serine	3.06 ± 0.01^c^	3.17 ± 0.00^b^	3.53 ± 0.01^a^	
Glutamic acid	17.87 ± 0.03^c^	20.23 ± 0.03^b^	22.46 ± 0.06^a^	
Total NEAAs	41.79	46.95	53.65	
Conditionally essential amino acids				
Proline	2.18 ± 0.00^c^	2.68 ± 0.00^b^	3.75 ± 0.00^a^	
Glycine	2.37 ± 0.01^c^	2.63 ± 0.00^b^	3.02 ± 0.00^a^	
Arginine	8.28 ± 0.00^c^	8.66 ± 0.01^b^	9.66 ± 0.01^a^	
Cysteine	1.68 ± 0.00^c^	1.79 ± 0.00^b^	2.02 ± 0.00^a^	
Tyrosine	1.97 ± 0.01^c^	2.09 ± 0.00^b^	2.34 ± 0.01^a^	
Histidine	1.93 ± 0.01^c^	2.50 ± 0.06^b^	2.94 ± 0.00^a^	2.1
Total	18.41	20.35	23.73	
Essential amino acids				
Lysine	0.312 ± 0.01^c^	0.363 ± 0.02^b^	0.405 ± 0.01^a^	4.2
Threonine	3.02 ± 0.01^c^	3.35 ± 0.00^b^	3.93 ± 0.00^a^	2.8
Valine	1.08 ± 0.01^c^	1.25 ± 0.00^b^	1.64 ± 0.00^a^	4.2
Methionine	0.31 ± 0.00^c^	0.35 ± 0.01^b^	0.41 ± 0.01^a^	2.2
Isoleucine	4.23 ± 0.00^c^	4.69 ± 0.00^b^	5.14 ± 0.01^a^	4.2
Leucine	3.83 ± 0.01^c^	4.08 ± 0.00^b^	5.04 ± 0.00^a^	4.2
Phenylalanine	3.27 ± 0.01^c^	3.57 ± 0.00^b^	4.25 ± 0.00^a^	2.8
Tryptophan	ND	ND	ND	
Total	16.052	17.653	20.815	33.9

Values are means (±SEM) of triplicate samples; means with different superscripts in the same row show significant difference (*P* < 0.05). ND, not detected; RMO, raw *M. oleifera* flour; GMO, germinated *M. oleifera* flour; FMO, fermented *M. oleifera* flour.

* FAO/WHO (1990).

**Table 4:** Chemical scores of essential amino acids of raw, germinated, and fermented *Moringa oleifera* seeds

Amino acid scores	FAO/WHO ref.	RMO	GMO	FMO
Lysine	5.8	5.34	6.21	7.07
Threonine	3.4	88.82	98.53	115.59
Valine	3.5	30.86	35.71	46.86
Methionine	2.2	14.09	15.91	18.64
Isoleucine	2.8	151.07	167.50	183.57
Leucine	6.6	58.03	61.82	76.36
Phenylalanine	2.8	116.79	127.50	151.79
Histidine	1.9	101.58	131.58	154.74
Tryptophan	1.1	0.00	0.00	0.00
Arginine	2	414.00	433.00	483.00
Total	33.9	88.23	96.43	111.45
First limiting amino acid		Lysine	Lysine	Lysine
Second limiting amino acid		Methionine	Methionine	Methionine

Values are means (±SEM) of triplicate samples; means with different superscripts in the same row show significant difference (*P* < 0.05). RMO, raw *M. oleifera* flour; GMO, germinated *M. oleifera* flour; FMO, fermented *M. oleifera* flour.

### Fatty acids of *M. oleifera* seed flour

The fatty acid composition of RMO, GMO, and FMO seeds are shown in Table 5. The most abundant fatty acid in the *M. oleifera* seed flour is linoleic acid, which ranged between 58.79 ± 0.02 g/100 g for RMO and 62.05 ± 0.01 g/100 g for FMO, while the least value was behenic acid and the value ranged from 0.13 ± 0.00 for GMO to 0.20 ± 0.06 g/100 g for the FMO sample. The high percentage of polyunsaturated fatty acid (PUFA) of the *M. oleifera* seed flour has some nutritional advantages. For instance, it is evident that high consumption of PUFA decreased the risk of coronary heart diseases in an affected population (Ferrier et al. 1995). Also, the high level of linoleic acid, the precursor of decohexanoic acid (DHA) and arachidonic acid, found in moringa seeds is an added advantage for the consumption of the seed, particularly for children. It is well known that DHA and arachidonic acid are critical for the growth and development of the nervous system in the first 6 months of life (Innis 1992). Industrially, the behenic acids found in *M. oleifera* seed flour make it useful as a solidifying agent in margarines and other foodstuffs containing solid and semisolid fat, thereby eliminating hydrogenation processes (FAO 2001). The PUFA/saturated (P/S) ratio ranged from 2.16 for RMO to 2.18 for FMO. Comparatively, the P/S ratios of GMO and FMO were lower than that of RMO; however, they were similar to the values reported for soybean (3.92), corn oil (4.65), and quinoa oil (4.90) (Vadivel and Janardhanan 2005). The P/S ratio therefore revealed that the *M. oleifera* seed flour is a relatively good source of PUFA.

**Table 5:** Fatty acid (% fatty acid) of raw, germinated, and fermented *Moringa oleifera* seed flour

Fatty acids	RMO	GMO	FMO
Saturated fatty acids			
Myristic acid	0.57 ± 0.00^a^	0.42 ± 0.01^b^	0.44 ± 0.01^b^
Palmitic acid	13.48 ± 0.00^c^	14.01 ± 0.01^b^	14.50 ± 0.00^a^
Stearic acid	12.62 ± 0.01^b^	13.57 ± 0.03^a^	13.52 ± 0.01^a^
Behenic acid	0.16 ± 0.00^a^	0.13 ± 0.00^a^	0.20 ± 0.06^a^
Arachidic acid	ND	ND	ND
Total	26.83 ± 0.01	28.13 ± 0.05	28.66 ± 0.08
Poly unsaturated fatty acids			
Linolenic acid	0.18 ± 0.00^b^	0.13 ± 0.01^c^	0.32 ± 0.01^a^
Linoleic acid	58.79 ± 0.02^c^	60.70 ± 0.01^b^	62.05 ± 0.01^a^
Arachidonic acid	ND	ND	ND
Total	58.97 ± 0.00	60.83 ± 0.02	62.37 ± 0.02
Mono unsaturated fatty acids			
Palmitoleic acid	0.36 ± 0.00^a^	0.26 ± 0.00^b^	0.22 ± 0.00^c^
Oleic acid	13.18 ± 0.00^a^	10.25 ± 0.03^b^	8.32 ± 0.02^c^
Total	13.54 ± 0.00^a^	10.51 ± 0.03^b^	8.54 ± 0.02^c^
P:S	2.19	2.16	2.18

Values are means (±SEM) of triplicate samples; means with different superscripts in the same row show significant difference (*P* < 0.05). ND, not detected; RMO, raw *M. oleifera* flour; GMO, germinated *M. oleifera* flour; FMO, fermented *M. oleifera* flour; P, phosphorus; S, sulfur.

### Antinutrient and phytochemical component of *M. oleifera* seed

The phytochemical component of RMO, GMO, and FMO seed flour is presented in Table 6. The phytochemical components in moringa were as follows: tannin (146.67 ± 3.33–241.67 ± 1.67 mg/100 g), phytate (28.33 ± 1.67–78.33 ± 1.67 mg/100 g), phenolics (23.00 ± 1.00–40.00 ± 0.00 mg/100 g), alkaloids (12.33 ± 0.17–17.33 ± 0.17), while others were flavonoids (5.00 ± 0.02–5.50 ± 0.01 mg/100 g), saponin (7.50 ± 0.00–9.83 ± 0.17 mg/100 g), and terpenoids (20.00 ± 0.11–27.50 ± 0.21 mg/100 g). The study showed that the phytochemicals in germinated and fermented moringa seed flour samples were lower, except in terpenoids, than that of raw moringa seed flour. This observation shows that fermentation processing techniques significantly reduced the phytochemical components of moringa seeds than in the germination processing technique and in the unprocessed sample. An earlier study has reported that processing methods like germination and fermentation influenced the phytochemical compositions of processed food materials (Soetan 2008). Comparatively, the alkaloid content of moringa seed flour samples was lower than that of the upper limit of 60 mg/100 g recommended for a safe feed (McDonald et al. 1995). Tannin contents of the samples were lower than those reported for groundnut seeds (450.00 mg/100 g; Fasoyiro et al. 2006), sorghum grains (280.00 mg/100 g; Elemo et al. 2001), and Cajanus cajan (550.00 mg/100 g; Ayodele and Kigbu 2005). Similarly, the saponin content of moringa sample was lower when compared with that of *M. utilis* (5.20 mg/100 g) (Seena 2006). It is evident that antinutrients and phytochemicals have both adverse and beneficial effects in humans (Soladoye and Chukwuma 2012). For example, phytic acid, lectins, phenolic compounds and tannins, saponins, enzyme inhibitors, cyanogenic glycosides, and glucosinolates reduce the bioavailability of certain nutrients and impair growth in children (Elemo et al. 2001; Dingynan et al. 2003). On the contrary, when phytic acid, lectins, and phenolic compounds and saponins were used at low levels, they exhibited hypoglycemic, hypocholesterolemic and anticancer properties (Yoon et al. 1983; Sidhu and Oakenful 1986; Thompson et al. 1988; Jariwalla et al. 1990; Oakenfull and Sidhu 1990). Flavonoids have also been reported to possess antioxidant, anti-inflammatory, and antihypertensive properties (Ayinde et al. 2007; Li-Weber 2009).

**Table 6:** Phytochemical/antinutritional factor (mg/100 g) of raw, germinated, and fermented *Moringa oleifera* seed flour

Phytochemicals	RMO	GMO	FMO
Tannins	241.67 ± 1.67^a^	181.67 ± 1.67^b^	146.67 ± 3.33^c^
Phytate	78.33 ± 1.67^a^	40.00 ± 0.00^b^	28.33 ± 1.67^c^
Phenolics	40.00 ± 0.00^a^	34.33 ± 0.67^b^	23.00 ± 1.00^c^
Alkaloids	17.33 ± 0.17^a^	15.33 ± 0.33^b^	12.33 ± 0.17^c^
Flavonoids	5.50 ± 0.01^a^	5.50 ± 0.00^a^	5.00 ± 0.02^b^
Saponins	9.83 ± 0.17^a^	8.00 ± 0.29^b^	7.50 ± 0.00^b^
Terpenoids	20.00 ± 0.11^c^	27.50 ± 0.21^a^	25.00 ± 0.13^b^

Values are means (±SEM) of triplicate samples; means with different superscripts in the same row show significant difference (*P* < 0.05). RMO, raw *M. oleifera* flour; GMO, germinated *M. oleifera* flour; FMO, fermented *M. oleifera* flour.

### Functional properties of *M. oleifera* seed flour

The functional properties of RMO, GMO, and FMO seed flour are shown in Table 7. The functional properties of moringa seed flour ranged as follows: loose bulk density, 0.42 ± 0.01–0.45 ± 0.00 g/mL; pack bulk density, 0.16 ± 0.00–0.64 ± 0.01 g/mL; foaming capacity, 25.93 ± 0.41–37.70 ± 0.40; swelling capacity, 1.27 ± 0.03–1.50 ± 0.06, and WAC 80.33 ± 0.33–141.00 ± 1.00 g/mL. The foaming capacity, swelling capacity, and WAC of FMO were significantly higher than that of germinated and raw moringa seed flour samples (*P* < 0.05) except in bulk density. These values were either higher or lower than the reports of other researchers, for instance, bulk density of moringa seed flour was higher than soybean flour (0.38 g/mL) (Edema et al. 2005), but comparable to that of bambara groundnut (0.60–0.75 g/mL) (Onimawo and Egbekun 1998). The bulk density of flour samples influences the amount and strength of packaging material, energy density, texture, and mouth feel (Udensi and Okoronkwo 2006). For WAC, the value was high in moringa compared with soybeans (1.12 g/100 g; Alfaro et al. 2004) and mucuna seed flour (1.2–2.0 g/100 g; Adebowale et al. 2005). The high WAC observed in moringa seed flour could be due to the high protein content of the seed four, which has high affinity for water molecules (Yusuff et al. 2008).

**Table 7:** Functional properties of raw, germinated, and fermented *Moringa oleifera* seed flour

Parameters	RMO	GMO	FMO
Loose bulk density (g/mL)	0.45 ± 0.00^a^	0.42 ± 0.01^b^	0.44 ± 0.00^a^
Packed bulk density (g/mL)	0.63 ± 0.01^ab^	0.61 ± 0.00^b^	0.64 ± 0.01^a^
Foaming capacity (%)	25.93 ± 0.41^c^	29.63 ± 0.26^b^	37.70 ± 0.40^a^
Swelling capacity (%)	1.33 ± 0.03^b^	1.27 ± 0.03^b^	1.50 ± 0.06^a^
Water absorption capacity (g/mL)	80.33 ± 0.33^b^	80.33 ± 0.33^b^	141.00 ± 1.00^a^

Values are means (±SEM) of triplicate samples; means with different superscripts in the same row show significant difference (*P* < 0.05). RMO, raw *M. oleifera* flour; GMO, germinated *M. oleifera* flour; FMO, fermented *M. oleifera* flour.

## Conclusion

In conclusion, this study established that germination and fermentation processing methods improved the essential amino, fatty acids, and phytochemical composition of *M. oleifera* seed flour samples. However, fermentation increased essential amino acids and essential fatty acids with low antinutrient composition. In view of these, moringa seed flour could be incorporated into human diet, particularly during infancy, to prevent or reduce protein-energy malnutrition.
